# Economic Burden of Cervical Cancer in Bulgaria

**DOI:** 10.3390/ijerph20032746

**Published:** 2023-02-03

**Authors:** Hristina Lebanova, Svetoslav Stoev, Emilia Naseva, Violeta Getova, Wei Wang, Ugne Sabale, Elina Petrova

**Affiliations:** 1 Faculty of Pharmacy, Medical University-Pleven, 5800 Pleven, Bulgaria; 2 Faculty of Public Health “Prof. Tsekomir Vodenicharov, MD, DSc”, Medical University of Sofia, 1527 Sofia, Bulgaria; 3 Faculty of Pharmacy, Medical University of Sofia, 1000 Sofia, Bulgaria; 4 Center for Observational and Real-World Evidence, Merck & Co., Inc., Rahway, NJ 07065, USA; 5 Center for Observational and Real-World Evidence, Merck Sharp & Dohme, 11330 Stockholm, Sweden; 6 Market Access, MSD, 1407 Sofia, Bulgaria

**Keywords:** cervical cancer, cost of illness, economic burden

## Abstract

Bulgaria is among the European Union (EU) countries with the highest burden of cervical cancers and life expectancy below the EU average. The majority of cervical cancer cases (more than 95%) are caused by the human papillomavirus (HPV). The aim of this retrospective, cost of illness study is to identify direct healthcare costs of cervical cancer in Bulgaria from the payer perspective and to calculate indirect costs and the associated years of life lost. Costs data were sourced from the National Health Insurance Fund from January 2018 to December 2020. Years of life lost were calculated based on the country and gender-specific life expectancy. Indirect costs due to productivity loss were calculated using the human capital approach. The total treatment costs for 3540 patients with cervical cancer are EUR 5,743,657 (2018), EUR 6,377,508 (2019), and EUR 6,751,182 (2020). The costs associated with drug acquisition and administration accounted for the majority (63%) of total healthcare costs followed by hospital management costs (14%). An estimated total of 20,446 years of life were lost due to cervical cancer for the period 2018–2020. The costs of productivity losses are estimated at EUR 7,578,014. Our study showed that the economic burden of cervical cancer in Bulgaria is substantial. Focus on cervical cancer prevention via vaccination against the human papillomavirus, timely screening, early diagnosis, and higher vaccine coverage rates could reduce its economic burden in Bulgaria.

## 1. Introduction

Cervical cancer (CC) is the fourth most common type of cancer, diagnosed in female patients, and a leading cause of mortality among women. CC accounts for 6.5% of all malignancies in the female population. In 2020, an estimated 604,127 women were diagnosed with cervical cancer worldwide and about 342,000 women died from the disease [1,2]. Cervical cancer is the most commonly diagnosed cancer in 23 countries and is the leading cause of cancer-related deaths in 36 countries. The vast majority of these countries are in sub-Saharan Africa, Melanesia, South America, and South-Eastern Asia [3]. Moreover, there is a significant difference in incidence rates between countries in Western and Eastern Europe [4]. The human papillomavirus (HPV) is established as a leading cause of cervical cancer [5]. HPV vaccines are generally included in routine vaccination programs in developed countries with a target vaccination rate of 90% of girls fully vaccinated with the HPV vaccine by the age of 15 in Europe by 2030 [4,6]. However, cervical cancer is still a major public health problem, even in developed countries: 54,517 newly diagnosed cases of invasive cervical cancer are registered each year in Europe and 24,874 women die each as a consequence of cervical malignancies [7]. Cervical cancer is the second leading cause of cancer deaths for women between 15 and 44 years in Bulgaria [8]. Bulgaria is among the countries with the lowest health expenditures in the European Union (7.3% of GDP in 2018, 7.1% in 2019, and 8.5% in 2020) [9].

The prevalence of cervical cancer in Bulgaria is increasing according to the data from the National Statistical Institute, with increasing prevalence rates ranging from 15,691 cases in 2017 to 16,006 in 2019 [10,11]. The five years’ net survival from cervical cancer in Bulgaria is below the average for the EU (49.2% (95% CI 47.6–50.7) for 2000–2004 and 54.8% (95% CI 53.3–56.3) for 2010–2014) [12].

HPV is a group of more than 200 related viruses. Sexually transmitted HPV types fall into two groups, low risk and high risk. Low-risk HPVs mostly cause no disease. However, a few low-risk HPV types can cause warts on or around the genitals, anus, mouth, or throat. High-risk HPVs can cause several types of cancer. There are about 14 high-risk HPV types including HPV 16, 18, 31, 33, 35, 39, 45, 51, 52, 56, 58, 59, 66, and 68. Seven of these, HPV 16, 18, 31, 33, 45, 52, and 58, are responsible for most HPV-related cancers [13].

HPV 16/18’s attributable fraction among HPV+ cases is 72.8% (70.8–74.7) and HPV 16/18/31/33/45/52/58 are estimated to be responsible for 89.0% (95% CI: 87.5–90.3) of cases in Europe [14,15]. According to a Bulgarian study, close to 30% of all women between 15 and 54 years of age are infected with at least one HPV subtype with the highest prevalence in women between 15 and 34 years of age [5]. The most common subtype is HPV 16, followed by HPV subtypes HPV 56 and HPV 33. Interestingly HVP 18 was not detected in this study [16]. Another study found a very low rate of HPV 18 among women diagnosed with cervical cancer [17]. The most common low-risk HPV subtypes in women between 15 and 34 are HPV 16, HPV 11, and HPV 42 [16].

Vaccination against HPV and screening and treatment of pre-cancer lesions is a cost-effective way to prevent cervical cancer [1]. HPV vaccination is estimated to prevent up to 90% of HPV-related cancers [13]. There are studies proving cost-effectiveness of the vaccination prevention even with an estimated efficacy of 70%. Part of the key prerequisites for such conclusions is the combination of vaccine prevention and HPV screening [18].

Since 2012, the HPV vaccine has been recommended and fully funded by the Bulgarian Government for girls 12–13 years old [19]. In 2021, the cohort was extended to ages 10–13 [7]. However, the target vaccination coverage rate of 75% was never achieved with vaccine coverage rates (VCR) for the last 6 years being below 10% [20]. These data are very alarming, especially if no improvement in screening and treatment strategies is achieved [21].

Cervical cancer imposes a considerable economic burden on society and individuals [5]. Understanding the clinical and economic burden of the disease is crucial for public health policy makers in the course of the decision-making process and budget planning [22]. Having in mind the current evidence of HPV vaccination preventing up to 90% of diseases attributed to this infection, a reasonable approach would be to evaluate the economic burden of cervical cancer to figure out an effective strategy for prioritization and allocation of resources for the prevention of cervical cancer [13]. The WHO also encourages decision makers in European countries to reinforce actions in eliminating cervical cancer by using the available preventative tools [23]. The relative burden of cervical cancer varies between different countries depending on local epidemiology, established type of disease management, and costs of treatment alternatives [24,25,26,27,28,29].

To our current knowledge, only one study has been conducted to calculate the burden of cervical cancer in Bulgaria [30]. Although widely referred to, the data are already outdated and may not reflect the current burden of cervical cancer in Bulgaria. The lack of recent country-specific burden of disease data makes informed governmental decisions for the improvement of public awareness, physician’s education, and immunization programs even harder to support. Data from other European countries suggest that the economic burden of cervical cancer is substantial [25,26,27,28,29]. The largest contributor to total healthcare costs is the inpatient care, accounting for 48% of costs for patients with cervical cancer [31].

Health services offered by the National Health Insurance Fund (NHIF) consist mainly of curative services and the majority of funds go to the hospital level and medicines’ acquisition [32]. Preventive services such as vaccination programs are funded by the Ministry of Health but in practice are vastly underrated [7]. Poor access to prevention services will shift and increase health services in secondary and tertiary care for cervical cancer [33]. An adequate and sustainable rate of HPV vaccination has the potential to decrease the burden and costs of HPV-related cancers over time as HPV vaccination has been found to be a cost-effective or even cost-saving strategy from both healthcare payer and societal perspectives [34,35,36]. Yet, due to poor prevention and low vaccination coverage rate in Bulgaria, morbidity and mortality of cervical cancer remain high [37].

Although the disease burden of cervical cancer in Bulgaria can be well described in terms of mortality, little is known about the direct economic burden of the disease and the burden expressed in the number of life years lost.

This cost of illness study aims to identify direct healthcare costs of cervical cancer in Bulgaria and to calculate indirect costs and years of life lost associated with cervical cancer.

## 2. Methods

### 2.1. Direct Costs

A prevalence-based cost of illness study was conducted based on data sourced from the Bulgarian national databases and registries for the period 2018–2020. For the identification of cervical cancer-specific healthcare resource utilization and associated costs, ICD-10 codes specific to cervical cancer (C53.0 (Malignant neoplasm of endocervix), C53.1 (Malignant neoplasm of exocervix), C53.8 (Malignant neoplasm of overlapping sites of cervix uteri), and C53.9 (Malignant neoplasm of cervix uteri, unspecified)) were used to identify the target population. The aggregated cost information was collected from the NHIF database without having access to patient-level information. Data were obtained based on ICD-10 codes for cervical cancer without any personal identifiers such as initials, age, city, etc.

Direct costs in the study include resource utilization for inpatient and outpatient care services related to the treatment and follow-up of cervical cancer patients. Publicly available and officially requested data for cervical cancer (ICD C53) management in Bulgaria were extracted from the National Health Insurance Fund (NHIF) database [38]. It provides information on the number of patients (by ICD-code), respective treatment, hospitalizations (by clinical pathway), and treatment costs (hospitalization, drug therapy, laboratory tests, ambulatory procedures, ambulatory visits, etc.). Data include primary care services, ambulatory health services, laboratory and imaging, hospitalization, treatments (chemotherapy, radiotherapy, brachytherapy, and palliation), clinical pathways, and the prevalence of cervical cancer. The clinical pathway is defined in the Bulgarian legislation as a system of requirements and guidelines for the behavior of different health professionals during diagnostic and treatment procedures of patients requiring hospitalization. Each hospitalization in the Bulgarian healthcare system is valued and paid to the provider according to the relevant clinical pathway.

The number of healthcare resources used associated with cervical cancer treatment per year was collected for the respective ICD C53 code (C53.0, C53.1, C53.8, and C53.9) and costed based on local tariffs within the National Framework Agreement 2020–2022 [32]. All identifiable costs are included in the estimate and were converted from Bulgarian lev (BGN) to Euro (EUR) using the fixed exchange rate of the Bulgarian National Bank (EUR 1 = 1.95583 BGN since 5 July 1997) [39]. All direct costs were estimated from the Bulgarian healthcare-payer perspective.

### 2.2. Indirect Costs

Country- and gender-specific life expectancy were sourced from the National Statistical Institute [40]. The years of life lost at the country level were calculated by summing the number of cervical cancer-specific deaths for a given age multiplied by the expected life years remaining at the mid-point for each age. The years of working life lost as a result of premature death due to cervical cancer were calculated by subtracting the age at death from the retirement age of 61 years. Years of life lost and years of working life lost were calculated for each age and then summed up. The number of deaths up to 61 years of age was then multiplied by the Gross Domestic Product per employed individual–current prices for the respective year and lost GDP per year were calculated. The human capital approach was used to estimate the indirect costs due to productivity loss. Lost productivity was defined as productivity loss as % of GDP incurred to society due to cancer-specific premature mortality.

### 2.3. Data Analysis and Statistical Methods

Data provided by the National Health Insurance Fund on drug therapy, inpatient, and outpatient costs were divided into categories (drug acquisition costs, inpatient, and outpatient costs) and were presented as a total healthcare cost for each year.

Data on drug consumption was presented according to the Anatomical Therapeutic Chemical (ATC) classification system where the active substances are divided into different groups according to the organ or system on which they act and their therapeutic, pharmacological and chemical properties. All drug costs were aggregated at ATC level 2 (pharmacological or therapeutic subgroup) and presented accordingly.

Data were analyzed and presented using descriptive statistics. The proportions of deaths up to 61 years of age were compared by using a *t*-test for two proportions. The results were considered as significant if p-values were smaller than 0.05. MS Office package (2019) as well as add-ons and SPSS v.22 were used.

### 2.4. Study Sample

The study sample consisted of the total number of patients diagnosed with C53.0, C53.1, C53.8, and C53.9 on treatment for the period 2018–2020 indexed in the NHIF database (Table 1). The number of patients who received inpatient treatment during the study period was consistent across the years. The most prevalent cervical cancer among hospital-treated patients was the malignant neoplasm of the exocervix (C53.1).

## 3. Results

### 3.1. Direct Costs

#### 3.1.1. Drug Acquisition Costs

The highest drug acquisition costs were observed in the C53.1 population, which corresponded with the number of patients diagnosed with malignant neoplasm of the exocervix (Table 2). An increase of 13% in drug acquisition costs is observed in 2019 vs. 2018. The 2020 costs remained at the 2019 levels.

The most commonly prescribed drug classes were the antineoplastic agents (ATC level L01), followed by the immunostimulants (ATC level L03) (Table 3). The increased drug acquisition costs in 2019 and 2020 vs. 2018 were due mainly to the increase in expenditures for antineoplastic agents (L01) and immunostimulants (L03).

#### 3.1.2. Drug Administration Costs

Drug administration costs were identified through clinical pathway P240 “Long-term systemic parenteral drug treatment of malignant solid tumors and related complications”. They were estimated at EUR 367,910.30 (2018), EUR 377,762.89 (2019), and EUR 442,040.46 (2020) representing 14%, 13%, and 15% of the total drug acquisition costs.

#### 3.1.3. Inpatient Costs

Inpatient costs consisted of radiotherapy costs, staging costs, and inpatient procedures costs. The relevant costs were extracted through the respective clinical pathways presented in Table 4. The largest share (55%) of the inpatient costs is associated with the costs of the procedures (mainly systemic radical excision of the lymph nodes). A significant increase was observed in robotic-assisted surgeries ranging from 39 procedures in 2018 to 105 in 2020 (an increase of 269%). The shares of the other inpatient cost categories remained stable. The expenditures on robotic-assisted surgeries were also the main driver for the increase in inpatient costs from EUR 1,505,268 in 2018 to EUR 2,047,723 in 2020. The higher inpatient costs were also a result of the higher prices for procedures set in the National Framework Agreement signed in 2020.

#### 3.1.4. Outpatient Costs

Outpatient procedure costs were also identified in the analysis. They consisted of monitoring the therapeutic response, determining a treatment plan, and PET/CT and SPEC/CT procedures. Costs of diagnostic tests (blood count, ultrasound tests, NMR, X-ray, etc.) were also included in this category (Table 5). Between 2018 and 2020, an increase of 3% was recorded. The 10% decrease in outpatient physician visits was mainly caused by COVID-19-related restrictions in 2020.

#### 3.1.5. Total Direct Costs

**Figure 1:**
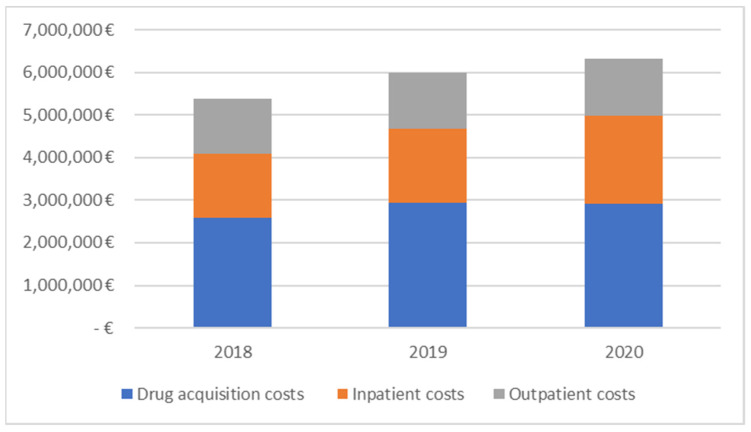
Distribution of healthcare costs among categories (2018–2020).

The direct costs of cervical cancer in Bulgaria range from EUR 5,375,747 in 2018 to EUR 6,309,141 in 2020 (Table 6). The observed increase is a result of a larger share of drug acquisition and inpatient costs (Figure 1).

### 3.2. Years of Life Lost and Indirect Costs

The human capital approach was used to estimate the indirect costs due to productivity loss. A 20% increase in the number of deaths by cervical cancer was recorded from 304 deaths in 2018 to 364 deaths in 2020. This trend is observed also in the group of deaths up to 61 years of age: a 30% increase in the number of deaths was observed in 2020 vs. 2018 (173 in 2020 vs. 137 in 2018). The proportion of deaths up to 61 years of age seems to decrease in 2019 and then increase again in 2020, but no significant difference is proven (*p* > 0.05).

For the study period, a total of 5092 years of working life were lost (1624 for 2018, 1507 for 2019, and 1961 for 2020). The mean years of life lost per person (±SD) are 20.7 (±11.2) for 2018, 20.1 (±10.8) for 2019, and 21.3 (±10.7) for 2020. The indirect costs associated with productivity losses range between 2 and 3 million euros per year based on the lost gross domestic product (GDP) per employed person (Table 7).

## 4. Discussion

Data from the NHIF database indicate that the overall costs (drugs, hospitalizations, outpatient, diagnostic) related to cervical cancer represent a significant burden to the Bulgarian healthcare system, with a total of more than EUR 6.7 million annually, which accounts for 0.27% of the total NHIF budget [41]. The largest share is attributed to drug acquisition and administration costs (62–64%) followed by inpatient treatment costs (17.59% in 2020). The findings are consistent with previous studies which found that chemotherapy costs are the main driver of cervical cancer expenditures [31,42]. Our results show that the increase in direct costs in 2019 vs. 2018 was 11% and in 2020 vs. 2019 it was 6%. The observed increase is not due to annual inflation, as it was 3.1% in 2019 and 1.7% in 2020. The increase in hospital management and outpatient costs in 2020 is partially due to the adoption of a new National Framework Agreement defining the costs of healthcare services [32]. An important aspect of the study results is the rather low diagnostic costs which are generally due to high-rate out-of-pocket costs in this category–data that are not present in the NHIF databases [43,44].

It is difficult to directly compare the economic burden of cancer in Bulgaria (and cervical cancer in particular) with previously published data from other European countries due to differences in incidence and prevalence rates [4]. An analysis by Hofmarcher et al. outlines other factors such as differences related to the economic strength of the countries (GDP per capita), drug pricing systems, population earning levels reflecting productivity losses, etc. [45].

The decrease in the number of newly diagnosed cervical cancer patients (844 in 2020 vs. 892 in 2019) is likely due to restricted access to the healthcare system at the beginning of the COVID-19 pandemic than to the vaccination coverage rates (VCR) [46]. A considerable decrease is observed also in the number of outpatient visits in 2020, which is inevitably influenced by the COVID-19 pandemic and the associated restricted access to primary healthcare services [47]. Moreover, during the first year of the pandemic, much of the resources in the healthcare system were directed toward the management of the COVID-19 infection distribution.

A previously published study assessed an estimated annual burden of cervical cancer in Europe including Bulgaria [48]. The study reported that in 2013, the estimated mean number of new HPV-related cervical cancers in Bulgaria was 1094 (95% CI 1029–1159) with 974 (95% CI 913–1035) attributable to HPV16/18/31/33/45/52/58 [48]. The estimated mean annual number of cervical cancer cases in Bulgaria aligns with the number of cases reported in our study.

Our study showed that the cumulative direct costs of cervical cancer amount to EUR 17.68 million for 2018–2020. Considering that 89% of cervical cancer is linked to HPV, EUR 15.74 million (89%) can potentially be attributed to HPV [15]. These results highlight the importance of preventing HPV infection, which is also a major means of preventing cervical cancer cases.

The results of our study indicate that the number of years of life lost due to cervical cancer is gradually increasing. This increase is caused partly by the increased incidence (850 newly diagnosed patients in 2018 and 892 in 2019) and the potential effect of the COVID-19 pandemic in Bulgaria as the high level of excess mortality could be one of the reasons for the higher number of cervical cancer deaths registered in 2020 vs. 2019 (364 vs. 318) [49]. Late diagnosis, low vaccination coverage, and prevention are the other factors that affect mortality [7].

Currently, the HPV vaccine is recommended and fully funded by the Bulgarian Government for girls 12–13 years with the cohort being extended to 10–13 in 2021 [7]. However, the target vaccination coverage rate of 75% was never achieved, with VCR for the last 6 years being below 10% [7]. The VCR was 4% in 2018, which is one of the lowest VCRs in Europe [50]. Given the link between HPV infection and the number of cervical cancer cases as well as the associated costs, an adequate and sustainable rate of HPV vaccination is expected to significantly decrease the burden and costs of HPV-related cancers over time [48].

Numerous previously published studies prove that HPV vaccination is essential for cervical cancer elimination [15,48,51,52]. The addition of HPV31/33/45/52/58 to the current vaccine types currently available in Bulgaria (HPV 16/18/6/11) could prevent up to 90% of cervical cancers attributed to oncogenic HPV types [15]. Extrapolated to Bulgarian data, this could lead to 760 cases yearly if the current incidence rate is sustained. A modeling study from Sweden estimated that when younger birth cohorts no longer transmit HPV to women > 35 years of age, the HPV infection will no longer be sustained among older women [52]. Using Swedish population data, Dillner et al. estimated that at 18 years of age, the monthly incidence of HPV16 is 1.6%, whereas, above 35 years of age, it is <0.1%. Based on R0 values, the infections will rapidly disappear from women older than 35 years of age if the older population is no longer fed with new infections from the younger population, thus underlying the importance of HPV vaccination in younger ages [52]. Previously published cost-effectiveness studies consistently concluded that adolescent female-only, population-based HPV vaccination programs are cost-effective compared with cervical cancer screening alone [35,36,53]. Moreover, gender neutral vaccination was found to further decrease the cumulative incidence of HPV-related diseases and is found to be a cost-effective strategy compared to female-only programs [54].

To conclude, health education as well as the development and implementation of prevention strategies in children and young adults are key to maintaining public health and economic sustainability [55].

## 5. Limitations

Our study has several limitations that merit acknowledgment. First, data on costs are provided by the National Health Insurance Fund, which provides data from the public sector only. The results do not include out-of-pocket patients’ costs for outpatient visits, diagnostic tests, and follow-ups, which are suspected to be considerable. This makes our estimates conservative as the true economic burden of cervical cancer in Bulgaria is expected to be higher.

Second, it is impossible to measure the effect of COVID-19 on C53-related deaths in 2020, thus the suspicion of overestimation of years of life lost and productivity loss for 2020.

## 6. Conclusions

The economic burden of cervical cancer in Bulgaria is considerable, primarily driven by drug acquisition and administration costs followed by inpatient treatment costs. Focus on cervical cancer prevention, timely screening, early diagnosis, and higher HPV vaccine coverage rates could reduce the economic burden of cervical cancer in Bulgaria.

## Figures and Tables

**Table 1:** Study sample.

Year	2018	2019	2020
Patients indexed in the database (N)	1194	1214	1132
Patients in inpatient treatment (N)	916	904	937
C53.0-N (%)	259 (28.20%)	219 (24.23%)	220 (23.48%)
C53.1-N (%)	428 (46.78%)	472 (52.21%)	537 (57.31%)
C53.8-N (%)	103 (11.26%)	112 (12.39%)	86 (9.18%)
C53.9-N (%)	126 (13.77%)	101 (11.17%)	94 (10.03%)

Source: National Health Insurance Fund, data on file.

**Table 2:** Drug acquisition costs 2018–2020.

	2018	2019	2020
C53.0	EUR 798,688.28	EUR 561,907.84	EUR 555,624.44
C53.1	EUR 1,050,666.20	EUR 1,704,137.42	EUR 1,707,315.97
C53.8	EUR 288,619.32	EUR 324,911.68	EUR 313,153.13
C53.9	EUR 436,603.60	EUR 339,557.72	EUR 348,601.33
Total	EUR 2,574,577.39	EUR 2,930,514.67	EUR 2,924,694.87

**Table 3:** Drug acquisition costs by class.

	2018	2019	2020
B03 (antianemic preparations)	EUR 31,974.58	EUR 41,298.87	EUR 49,082.88
H02 (corticosteroids for systemic use)	EUR 3512.06	EUR 1120.80	EUR 1039.06
L01 (antineoplastic agents)	EUR 2,313,134.90	EUR 2,606,511.40	EUR 2,633,022.18
L03 (immunostimulants)	EUR 153,031.71	EUR 208,452.83	EUR 179,339.12
M05 (drugs for treatment of bone diseases)	EUR 8116.77	EUR 11,910.69	EUR 6813.92
N02 (analgesics)	EUR 64,797.37	EUR 50,789.60	EUR 47,320.74
V03 (all other therapeutic products)	EUR -	EUR 10,345.50	EUR 8076.96
Missing	EUR 10.00	EUR 84.98	EUR -
Total	EUR 2,574,577.39	EUR 2,930,514.67	EUR 2,924,694.87

**Table 4:** Inpatient costs.

Description of Procedures **	2018		2019		2020	
	N	Costs (EUR, %)	N	Costs (EUR, %)	N	Costs (EUR, %)
Radiotherapy costs						
High-tech radiotherapy for oncological and non-oncological diseases 3 days without radiation chemotherapy (P250.1)	461	EUR 436,055.28 (65%)	524	EUR 495,646.35 (71%)	423	EUR 420,657.73 (63%)
Intensity-modulated radiotherapy for oncological and non-oncological diseases hospital stay 5 days or not less than 5 procedures (P251.1)	173	EUR 238,824.44 (35%)	145	EUR 200,170.77 (29%)	179	EUR 248,937.79 (37%)
Total costs radiotherapy	EUR 674,879.72		EUR 695,817.12		EUR 669,595.52	
Staging costs						
Diagnostic procedures for staging and assessment of the therapeutic response in patients with malignant solid tumors and hematological diseases with CT of at least two zones or bone marrow examination with ICD-code 41.31 in persons over 18 years (P241.3)	0	EUR 0.00 - *	402	EUR 82,215.73	789	EUR 190,812.60
Inpatient procedures costs						
Non-radical hysterectomy (P160)	36	EUR 20,247.16 (2%)	37	EUR 20,809.58 (2%)	24	EUR 15,117.88 (1%)
Radical removal of female genitals (P161)	104	EUR 58,491.79 (7%)	87	EUR 48,930.63 (5%)	87	EUR 68,600.75 (6%)
Lower access surgical interventions to remove disease changes or invasive examination of the female genitalia (P163)	536	EUR 90,437.31 (11%)	566	EUR 95,499.10 (10%)	470	EUR 98,862.38 (8%)
Systemic radical excision of lymph nodes (pelvic and/or paraaortic and/or inguinal) as a stand-alone intervention or combined with radical removal of female genitals. Pelvic exenteration (P167)	446	EUR 501,679.59 (60%)	426	EUR 479,182.75 (50%)	415	EUR 574,175.67 (48%)
Robot-assisted gynecological surgery for malignancies (P168)	39	EUR 159,523.07 (19%)	78	EUR 319,046.13 (33%)	105	EUR 430,558.89 (36%)
Total inpatient procedures costs	EUR 830,378.92		EUR 963,468.19		EUR 1,187,315.57	
Total inpatient costs	EUR 1,505,258.64		EUR 1,741,501.04		EUR 2,047,723.69	

* no costs were reported by the NHIF. ** codes according to the National Framework Agreement [32].

**Table 5:** Outpatient costs.

Description of Procedures **	2018		2019		2020	
	N	Cost (EUR, %)	N	Cost (EUR, %)	N	Cost (EUR, %)
Outpatient procedures costs						
Systemic drug treatment of malignant solid tumors and haematological diseases (A06)	710	EUR 54,452.59 (6%)	1049	EUR 80,451.78 (9%)	1113	EUR 85,360.18 (9%)
Outpatient follow-up/medical examination for malignant diseases and congenital haematological diseases (A07)	0	EUR -	0	EUR -	0	EUR -
Monitoring of the therapeutic response in patients on home treatment with targeted oral antitumor therapy and oral chemotherapy (A08)	0	EUR -	0	EUR -	1	EUR 127.82 (0.01%)
Positron emission tomography with computed tomography (PET/CT) (PET/CT) (A35)	174	EUR 6227.54 (1%)	177	EUR 6631.97 (1%)	100	EUR 3936.95 (0.42%)
Single-photon emission computed tomography with computed tomography-SPECT/CT on a hybrid scanner (A36)	772	EUR 789,434.66 (92%)	960	EUR 785,344.33 (89%)	1032	EUR 844,245.15 (89%)
Determining a treatment plan and monitoring the therapeutic response in patients receiving expensive drugs under Art. 78, para 2 of the Health Insurance Act (A37)	69	EUR 12,347.70 (1%)	70	EUR 12,974.03 (1%)	63	EUR 12,401.38 (1%)
Total outpatient costs	EUR 862,462.49		EUR 885,402.11 €		EUR 946,071.49	
Diagnostic tests						
Blood count, at least eight or more of the following indicators: haemoglobin, erythrocytes, leukocytes, haematocrit, platelets, MCV, MCH, MCHC (01.01)	1071	EUR 1084.24 (8%)	1040	EUR 1052.85 (7%)	930	EUR 1093.65 (7%)
Ultrasound diagnosis of abdominal and retroperitoneal organs (06.34)	234	EUR 1647.47 (13%)	201	EUR 1415.14 (9%)	200	EUR 1585.00 (10%)
Nuclear magnetic resonance (10.02)	35	EUR 4033.76 (31%)	50	EUR 5762.52 (37%)	45	EUR 5636.99 (37%)
Computed axial or spiral tomography (10.01)	142	EUR 5586.11 (43%)	177	EUR 6962.97 (44%)	139	EUR 6396.26 (42%)
X-ray examination of the oesophagus, stomach * (06.37)	0	EUR -	0	EUR -	0	EUR -
Radiography of the pelvis (06.33)	7	EUR 49.28 (0.38%)	6	EUR 42.24 (0.27%)	7	EUR 55.48 (0.36%)
Cytological examination of two cytosmear samples from female genitals (07.09)	111	EUR 505.11 (4%)	110	EUR 500.55 (3%)	111	EUR 539.16 (4%)
Total diagnostic costs	EUR 12,905.97		EUR 15,736.27		EUR 15,306.55	
Follow up costs						
Physicians’ outpatients visits (A07)	6327	EUR 420,542.69	6418	EUR 426,591.27	5647	EUR 375,344.48
Total outpatient costs	EUR 1,295,911.15		EUR 1,327,729.65		EUR 1,336,722.51	

* no costs were reported by NHIF. ** codes according to the National Framework Agreement [32].

**Table 6:** Total healthcare costs of cervical cancer (2018–2020).

	2018	2019	2020
Drug acquisition costs	EUR 2,574,577.39	EUR 2,930,514.67	EUR 2,924,694.87
Inpatient costs	EUR 1,505,258.64	EUR 1,741,501.04	EUR 2,047,723.69
Outpatient costs	EUR 1,295,911.15	EUR 1,327,729.65	EUR 1,336,722.51
Total direct costs	EUR 5,375,747.18	EUR 5,999,745.36	EUR 6,309,141.07

**Table 7:** Indirect costs due to productivity loss.

	2018	2019	2020
N of deaths (all ages)	304	318	364
Average age of death (years, range)	62.33 (24–96)	62.97 (21–93)	61.47 (30–90)
N of deaths up to 61 years of age	137 (45.1%)	133 (41.8%)	173 (47.5%)
Years of life lost	6294.75	6401.93	7749.71
Mean YLL per person (±SD)	20.7 (±11.2)	20.1 (±10.8)	21.3 (±10.7)
Years of working life lost	1624	1507	1961
Mean years of working life lost per person (±SD)	11.9 (±7.9)	11.3 (±8.2)	11.3 (±7.5)
GDP per employed–current prices (EUR) *	EUR 15,965.23	EUR 17,420.61	EUR 17,767.83
Lost GDP (not produced GDP), EUR per year	EUR 2,187,236.80	EUR 2,316,941.70	EUR 3,073,835.04

* National Statistical Institute.

## Data Availability

Data presented in this study are available in the article.
